# Supplementary material for: Beta-Barrel Channel Response to High Electric Fields: Functional Gating or Reversible Denaturation?
Source: Int J Mol Sci. 2023 Nov 23;24(23):16655. doi: 10.3390/ijms242316655 (PMC10706840; doi:10.3390/ijms242316655)
Supplement: Supplementary file 1 [file ijms-24-16655-s001.zip › ijms-2666488-supplementary.pdf]

Supplementary Material

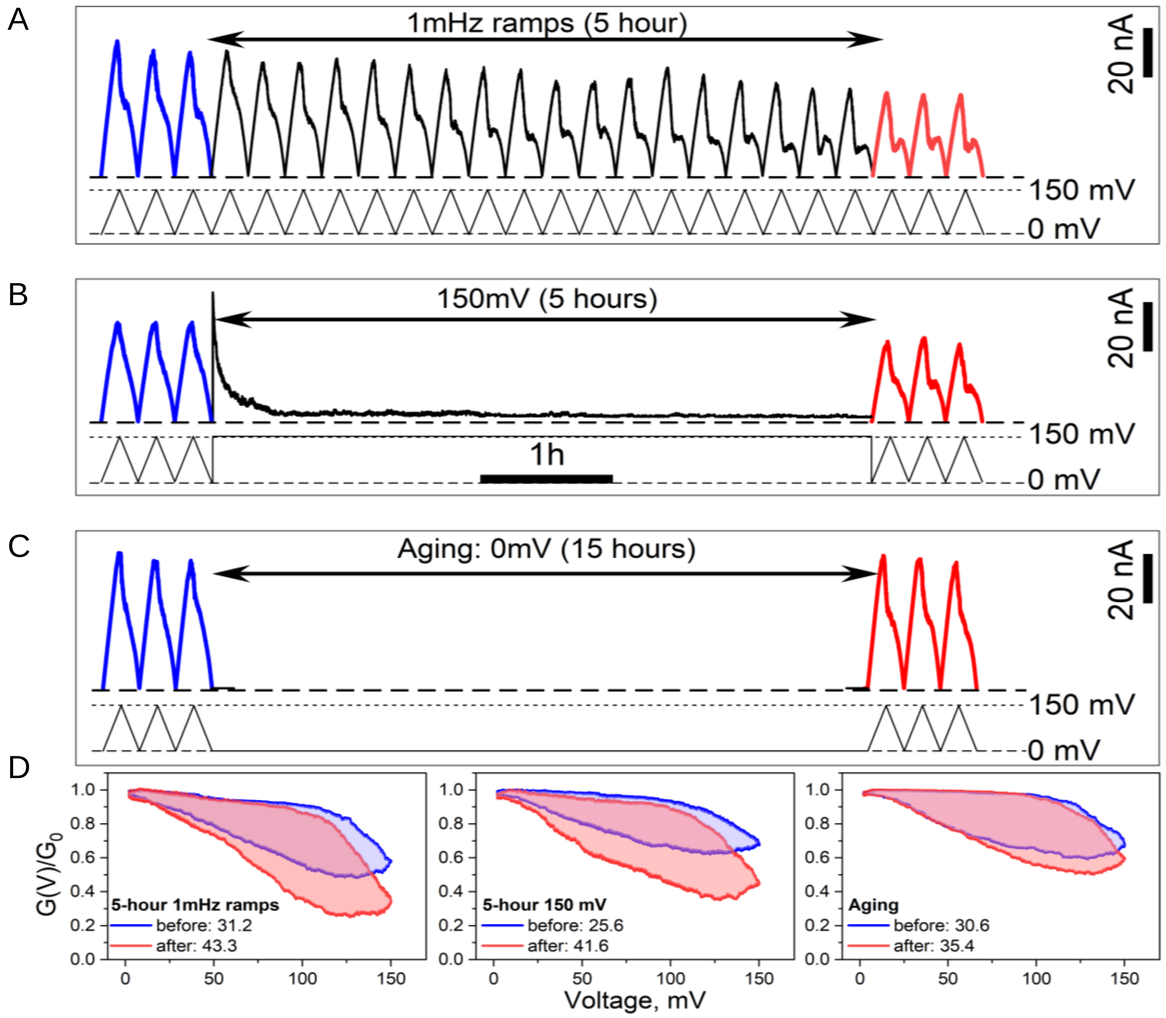

Fig. S1. OmpF voltage gating memory protocols. (A) Voltage Ramps: Raw data displaying ion current across a multichannel membrane in response to a series of 24 repeated voltage ramps from 0 to 150 mV, applied at a frequency of 1 mHz. **(B)** Voltage Clamp: Raw data illustrating ion current across a multichannel membrane, featuring three voltage ramps from 0 to 150 mV recorded both before and after a 5-hour application of 150 mV, all at a 1 mHz frequency. **(C)** Aging Effect: Raw data presenting ion current across a multichannel membrane, with three voltage ramps from 0 to 150 mV recorded both before and after a 15-hour application of 0 mV, all at a 1 mHz frequency. In panels (A-B), the blue and red voltage ramps represent the "before" and "after" ramps, respectively, which are further analyzed and depicted in panel (D). **(D)** Hysteresis Analysis: The left panel displays data from panel (A), the middle panel shows data from panel (B), and the right panel displays data from panel (C). Notice the variations in the areas enclosed by the hysteresis curves across all three experiments.

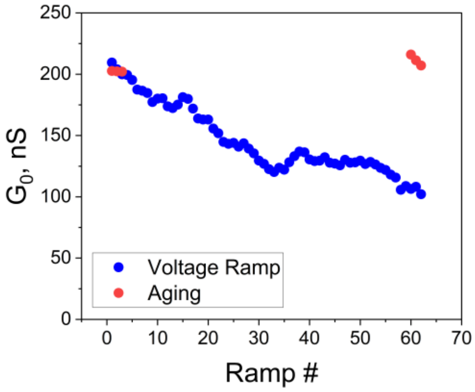

Fig. S2. **Impact of voltage ramp application and channel/membrane aging on membrane conductance**. The figure compares multichannel membrane conductance approximated to a 0 mV applied voltage for two independent experiments corresponding to protocol 1 and protocol 3, as explained in Fig. S1A and C, respectively. Notably, with the application of sixty-two consecutive 0 to 150 mV ramps at a 1mHz frequency, the number of fully open OmpF channels progressively decreases with each ramp in protocol 1, indicating that some channels remain closed due to voltage gating without reopening during the experiment. In contrast, for protocol 3, where only three ramps are measured before and after the membrane is maintained at 0 mV for fifteen hours, the multichannel conductance approximated to 0 mV remains relatively stable, suggesting that aging itself does not decrease the number of open channels in the membrane.

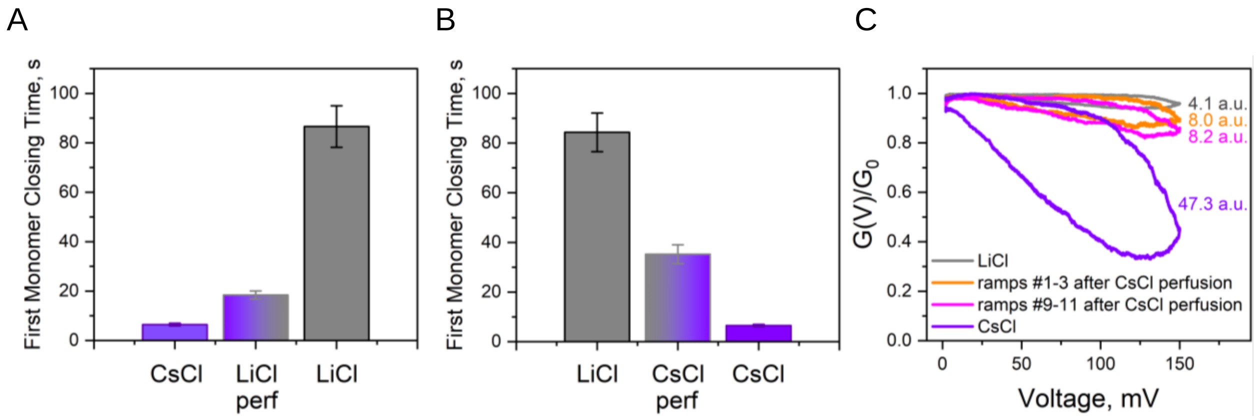

Fig. S3. The Hofmeister effect and "gating" memory in OmpF channels. (A) First monomer closing times when a single OmpF channel was incorporated into PLM in 1M CsCl solution (*violet column*) and then the solution was perfused with a tenfold volume of LiCl (*bicolor violet and gray column*). The data are compared with the single channel experiment performed in pure LiCl (*gray column*). **(B)** First monomer closing times when a single OmpF channel was incorporated into PLM in 1M LiCl solution (*gray column*) and then the solution was perfused with a tenfold volume of CsCl (*bicolor gray and violet column*). The data are compared with a single-channel experiment performed in pure CsCl (*violet column*). **(C)** 1 mHz 0 to 150 mV hysteresis curves recorded in 1M LiCl solutions (*gray*) and, for the same membrane, in solutions with a fifteenfold volume perfusion with CsCl. Specifically, it shows the behavior of the first three ramps (*orange*) and ramps #9-11 (*magenta*) following the solution exchange. While a moderate increase in the hysteresis area (4.1 a.u. in LiCl, 8.0 a.u. for ramps #1-3, 8.2 a.u. for ramps #9-11) was observed, indicating a gradual change in gating kinetics, it is noteworthy that the channels did not attain the rapid kinetics independently observed in CsCl (*violet*) (47.3 a.u.).
